# Digital Computer Matching of Tooth Color

**DOI:** 10.3390/ma3063694

**Published:** 2010-06-18

**Authors:** Won-suk Oh, John Pogoncheff, William J. O’Brien

**Affiliations:** Department of Biologic and Materials Sciences Division of Prosthodontics, University of Michigan School of Dentistry, 1011 North University, Ann Arbor, MI 48109, USA; E-Mails: pogonj@umich.edu (J.P.); wjobrien@umich.edu (W.J.O.)

**Keywords:** visual shade matching, digital photocolorimetric method, dental shade guide

## Abstract

This study aimed to determine the validity of the digital photocolorimetric (PCM) method in matching the color of human teeth. First, two Vitapan Classical shade guides, each containing 16 shade guide teeth, were visually shade matched, and digital photographs of each three pair of shade guide teeth were taken in a color matching booth. Secondly, visual shade matching of the upper central incisors of 48 subjects was performed by two prosthodontists independently in a chair, using the Vitapan Classical shade guide. The three closest shade guide teeth were visually selected and ranked in order of preference, for which digital photographs were taken under ceiling daylight-corrected fluorescent lighting. All digital images were analyzed on a computer screen using software to calculate the color difference between the reference tooth and other teeth in the same digital image. The percent color matching for the shade guide teeth and human teeth was 88% and 75%, respectively. There was no statistically significant difference in matching the tooth color between the shade guide teeth and human teeth. The digital PCM method is valid for the range of human teeth based on the Vitapan Classical shade guide. This method enhances communication with the laboratory personnel in matching the tooth color.

## 1. Introduction

Visual shade matching is a common method, in which a color standard from a commercially available dental shade guide is compared to and matched with the target tooth [1,2,3,4]. However, the visual shade selection varies, depending on the clinician’s color perception and experience, ambient light condition, background of the tooth, and the shade guide used [5,6,7]. Communication with a dental laboratory is another problem [8]. The technician does not usually see the patient and has to work on dentist’s written prescription as based on the shade guide used.

Spectrophotometric measurement methods employing computer calculations, based on color science and theory, allow quantitative evaluation [9,10]. This method is objective and appears to be more accurate; however, the quantitative spectrophotometric evaluation is limited to reading one point at a time. Additionally, there is a lack of standardization, high costs, and relatively low performance, with respect to agreements of the computer-aided devices. Incorrect color reading from the loss of a fraction of light entering the tooth, the so called “edge-loss error”, is a frequent shortcoming of contact-type devices [11].

Photographic images make it possible to evaluate several points, which can aid in determining the true shade of a tooth [1,8]. Due to advances in photography and computer technology, the use of a digital camera is now widespread for color imaging. The instrument is capable of recording digital data from an object and producing an image on a computer screen, when can then be transmitted via the Internet. Images produced via a digital camera may be analyzed using appropriate imaging software, enabling the collection of color values from the whole or parts of such images [12,13,14]. Shade matching that is based on digital imaging is convenient and less expensive than the use of spectrophotometers or colorimeters, and may provide the entire spectrum of color space for natural teeth. Additionally, digital imaging is recognized as an objective and efficient tool for communication with a dental laboratory. However, the validity of the method has yet to be proven.

The purpose of this study was to determine the validity of the digital photocolorimetric (PCM) method in matching the color of human teeth. The null hypothesis was that the digital PCM method would be consistent for both the full range of shade guide teeth and human teeth.

## 2. Results and Discussion

### 2.1. Experiment I—Matching of Duplicate Shade Guide Teeth and Photography

The percent correct matching of duplicate shade guide teeth by minimum color difference, ΔE*, with the computer program, was 88%, with an average ΔE* of 2.3 (SD = 0.7) between duplicate shade guide teeth. Two shades A1 and C1 were incorrectly mismatched with shades B1 and D2, respectively.

### 2.2. Experiment II (Clinical)—Visual Shade Matching of Human Teeth

The proportion of visually selected shade guide teeth matching ones with the lowest ΔE* against the target tooth by the digital PCM method was 75%, allowing for a tolerance of ΔE* of less than 3.0. For example, shade B1, if matched with A1, is an acceptable match since there is a clinical tolerance in shade matching [15].

The null hypothesis was not rejected. A t-test (α = 0.05) revealed no statistically significant differences between the rates of correct matching of duplicate shade guide teeth and the “agreement” for human teeth using the digital PCM method.

The selection of the three visually closest shade tabs in an order of preference by a dentist and subsequent digital photography, along with analysis on a computer screen using a digital matching instrument, can make a final match based upon the lowest ΔE*.

The experiment II showed that only half of the “best” shade guide teeth chosen by visual perception matched with the ones determined by the digital PCM method, but 75% of visual selection was clinically acceptable, as determined by the digital PCM method. A study of the raw data shows that observer A selected more shades in the “C” or “D” series, whereas observer B selected more from the “A” and “B” series. This may relate to the common, relatively low rate of inter-observer agreement, as noted in previous reports [3,4]. Thus, photographic shade matching based on digital technology and colorimetric assessment may minimize negative clinical outcomes associated with inaccurate and unreliable visual shade matching of the tooth, promote shade matching, and reduce possible errors of shade mismatches [1,12,13,14].

Communication with the dental laboratory could be improved by using the digital photographic image of the target tooth, along with the visually selected shade guide teeth. The clinical application of this method would involve the dentist taking a digital photograph of the patient’s target tooth, adjacent with his (or her) two closest visually matched shade guide teeth. The dentist or dental technician then displays this photograph on a computer screen and uses the Eye-One Match or a similar instrument to choose the closest shade for the final match.

## 3. Experimental Section

### 3.1. Experiment I—Matching of Duplicate Shade Guide Teeth and Photography

Two Vitapan Classical shade guides, A and B (Vita Zahnfabrik, Bad Säckingen, Germany), each of which has 16 shade guide teeth, were shade matched in a color matching booth (GTI ColorMatcher, GTI Graphic Technology, Newburgh, New York, USA) using D65 daylight (6,500 K), by an experienced laboratory ceramic technician, who demonstrated excellent matches in a color calibration test [1]. The shade guide teeth were arranged according to value from the lightest to the darkest according to the manufacturer, pair-matched, and taped in groups of three pairs of shade guide teeth on a white background.

**Figure 1:**
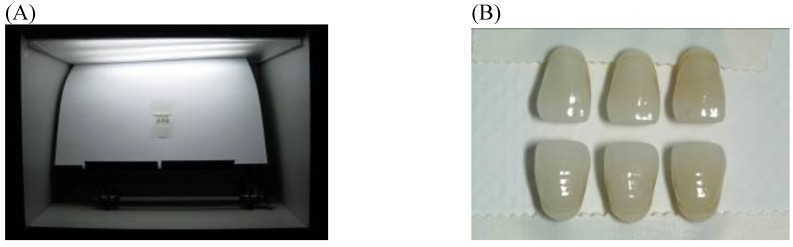
(A) A group of three paired shade guide teeth taken in a color matching booth. (B) Photocolorimetric shade matching of the paired shade tabs. Each of the vertical pairs are duplicates of three Vita Classical shade tabs.

Digital photographs of each three paired shade guide teeth were taken in the color matching booth (GTI ColorMatcher) shown in Figure 1. The Nikon D70s digital SLR camera (Japan) was set on manual mode (M) program, and used with a 105 mm macro lens at 1:1 magnification. The shutter speed was set at 125 second with the aperture of F20. A bilateral flash (R1 TTL Ring Light Flash, Nikon, Japan), at an angle of 45°, was fixed to the front of camera lens.

The photographic images were transferred from the camera to a computer, saved as JPG files, displayed on a computer screen, and measured with a probe of the Eye-One instrument (Eye-One Match, Gretag-MacBeth, Germany). A spot measurement for L*, a*, b* (Commission Internationale de l’Eclairage L* a* b* color system) was then taken of each tooth of shade guide A from the computer screen images and recorded in the software to serve as a reference. Then L*a*b* measurements were taken for each tooth of shade guide B images, at which time the software automatically calculated the color difference (ΔE*) between shade guide A and B.

The lowest ΔE* values were identified out of each three paired shade guide teeth to determine the validity of the digital PCM method in the full range of commercial shade guide teeth.

### 3.2. Experiment II (Clinical)—Visual Shade Matching of Human Teeth

A total of 48 volunteers, 26 men and 22 women, ages 20–70, were recruited as subjects for shade matching of the middle third of the right maxillary central incisors. Visual tooth shade matching was performed using the Vitapan Classical shade guide (Vita Zahnfabrik) of 16 shade guide teeth, which were arranged according to value from the lightest to darkest, according to the manufacturer.

**Figure 2:**
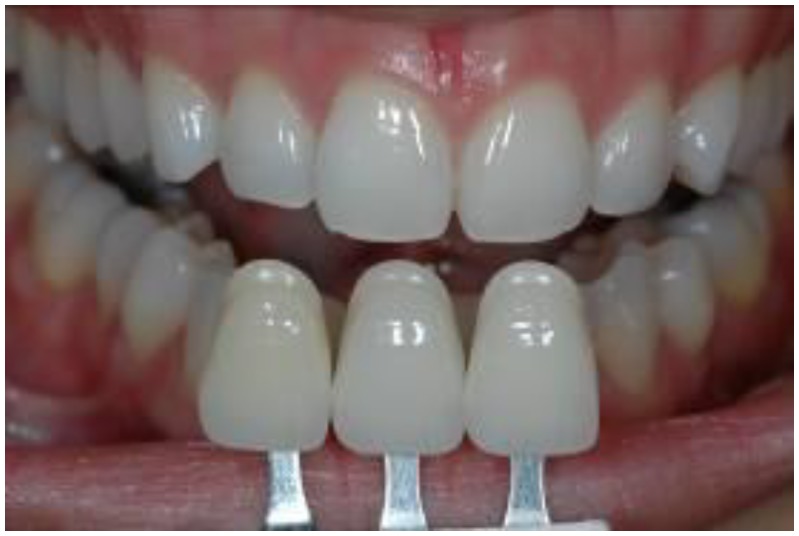
Visual shade matching of the three visually closest shade tabs of a commercially available shade guide against the upper right central incisor.

Two prosthodontists (Observers A and B), who showed high accuracy for matching shades in a color calibration test, independently chose three of the visually closest shade guide teeth to match the patient’s target tooth, and ranked them in order of best, second best, and third best matches (Figure 2) [4]. Digital photographs of the patients’ upper front teeth were taken with the three visually selected shade guide teeth that were directly placed incisal to the target tooth. The Nikon D70s digital SLR camera was set as in Experiment I. The position of each volunteer was standardized by sitting upright in a chair. Lip retractors were used to enable adequate lighting to enter the oral cavity. The 96 images (two observers x 48 volunteers) were taken and analyzed on a computer screen using software (Eye-One Match), as in Experiment I. The target tooth served as a reference for each shade tab in the same digital image. This process was repeated to compare the ΔE* between each shade tab selected as a possible match to the target tooth.

Three measurements were made on each target area of tooth and shade guide tabs and the average values of ΔE* between the target tooth and each shade guide tooth were obtained. The three shade guide tabs were rank ordered according to the average ΔE*, with the minimal ΔE* as the best matching shade tab.

The ΔE* between the best matching shade tabs selected by means of visual observation and the digital PCM method based on a digital image for each volunteer was calculated for Observers A and B. Percentage analyses were also calculated to determine the clinical acceptability of color differences. A ΔE* of less than 3.0 was defined as “agreement” [15].

### 3.3. Statistical Analysis

Statistical analysis was performed using a t-test (Statistica 2007, StatSoft Inc, Tulsa, OK, USA) to reveal significant differences in color matching between the duplicate shade guide teeth and visual shade matching of human teeth, using the photo-colorimetric method.

## 4. Conclusions

Tooth color matching using the digital PCM method was found to be valid with the use of the Vitapan Classical shade guide. This digital technology could provide an alternative accurate method of tooth color matching by enhancing communication with the laboratory personnel.
